# A rational approach to migraine diagnosis and management in primary care

**DOI:** 10.1080/07853890.2021.1995626

**Published:** 2021-10-29

**Authors:** Vincent T. Martin, Alexander Feoktistov, Glen D. Solomon

**Affiliations:** a Department of Internal Medicine, University of Cincinnati, Cincinnati, OH, USA; b Synergy Integrative Headache Center, Northfield, IL, USA; c Department of Internal Medicine, Wright State University, Dayton, OH, USA

**Keywords:** Migraine, diagnosis, acute therapy, preventive therapy, calcitonin gene related peptide (CGRP)

## Abstract

Migraine is a chronic neurologic disease estimated to affect approximately 50 million Americans. It is associated with a range of symptoms, which contribute to disability and substantial negative impacts on quality of life for many patients. Still, migraine continues to be underdiagnosed, undertreated, and optimising treatment for individual patients has proven difficult. As many migraine patients will be seen first in primary care settings, internists and other primary care providers are ideally positioned to improve diagnosis and migraine management for many patients. In this review, we discuss some of the challenges in diagnosing migraine and suggest strategies to overcome them, summarise the current understanding of migraine pathophysiology and clinical evidence on acute and preventive treatment options, and offer practical approaches to diagnosis and contemporary management of migraine in the primary care setting.

**Key messages**
- Migraine is a prevalent disease with substantial impact. Primary care providers are ideally positioned to improve care for migraine patients with streamlined approaches to diagnosis and management.
- A stepwise diagnostic approach to migraine involves taking a thorough headache history, excluding secondary headache, and identifying primary headache disorder using screening tools or ICHD-3 criteria.
- The FDA approved seven new migraine therapies from 2018 to 2020 (four monoclonal antibodies, two gepants, one ditan), expanding acute and preventive therapeutic options.

## Introduction

An estimated 112.7 million Americans suffer from headache disorders, including more than 47 million individuals experiencing migraine [1]. Although migraine can occur in all ages, it most heavily impacts, and is the leading cause of years lived with disability for, adults under age 50 [2]. Migraine peaks in prevalence for people, particularly women, in their thirties [1], with the American Migraine Prevalence and Prevention (AMPP) Study showing 24.4% of women and 7.4% of men age 30–39 have migraine [3]. The observation from multiple studies indicate that the change in homeostasis or environment, such as emotional stress, physical activity, disrupted sleep pattern, eating habits and various odours are common triggers for migraine [4–7].

A systematic review of 18 large-scale studies from 12 countries found that headache was the seventh most common patient-reported reason for visiting primary care [8]. A US study analysing ICD-9 codes estimated that headache disorders account for approximately four million primary care office visits annually, which is comparable to the number of visits for hypercholesterolaemia (4.6 million) [9]. Among patients presenting with headache in the primary care setting, migraine is the most common diagnosis; one study suggested that more than 90% of patients consulting for headache in primary care could be diagnosed with migraine or probable migraine (defined as migraine-like attacks lacking one of the features needed to fulfil all diagnostic criteria [10,11]). Migraine has an estimated prevalence of approximately one-third of patients seen in the primary care setting for any complaint in the US [12].

Studies have suggested that migraine is underdiagnosed and undertreated [13]. Large surveys show less than half of individuals with migraine-associated disability had seen a provider for their headache symptoms in the prior year [14,15]. Among individuals that do consult a physician for headache, only 87% of those with episodic migraine (EM; <15 headache days/month) and 25% of those with chronic migraine (CM; ≥15 headache days/month, of which ≥8 days have migraine features for >3 months) received an appropriate diagnosis [14,15].

Internists and other primary care providers are in a key position to provide timely diagnosis and optimal treatment for these patients. Given the likelihood of seeing migraine patients in primary care, we review some of the challenges in diagnosing migraine and discuss strategies to overcome them, provide an overview of the evolving understanding of migraine pathophysiology, and examine contemporary approaches to migraine management.

## Common barriers for proper migraine diagnosis and strategies to overcome them

**Figure 1.**
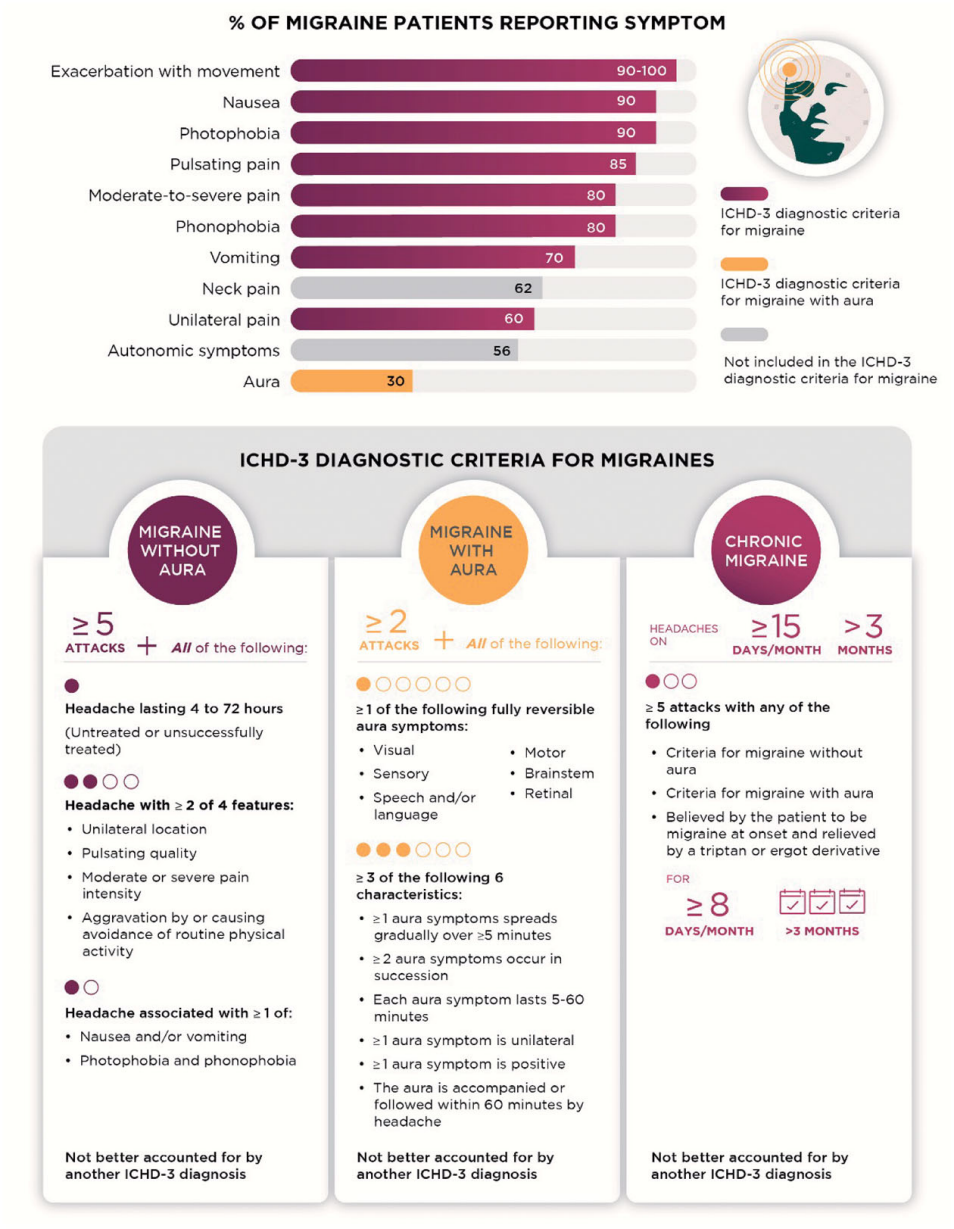
Variability of Typical Migraine Symptoms and overview ICHD-3 diagnostic criteria for migraine. The estimated frequency of the indicated symptoms among patients with migraine is shown [23–27]. Bar colour indicates if symptoms are included in the ICHD-3 diagnostic criteria for migraine (plum), migraine with aura (gold), or neither (grey). Lower panel shows diagnostic criteria for migraine without aura, migraine with aura, and CM [10].

Migraine is associated with a variety of symptoms that can differ among individuals and between attacks in the same individual [16], as shown in Figure 1. The International Classification of Headache Disorders (ICHD)-3 diagnostic criteria require a combination of the most common symptoms for migraine diagnosis (Figure 1), but patients do not need to exhibit all the features listed in the ICHD-3 criteria. Notably, migraine can be diagnosed in the absence of characteristic symptoms such as aura, throbbing, or severe pain, as not all these symptoms occur in all patients (Figure 1).

Additional symptoms including nasal congestion, rhinorrhea, and pain near the maxillary or frontal sinuses are also associated with migraine in some patients, although they often prompt inaccurate diagnoses of “sinus headache.” Likewise, headaches triggered by stress or psychological disorders can be misidentified as tension headaches, while studies suggest multiple headache types, including migraine, worsen with stress [17].

Further complicating migraine diagnosis is the observation that patients do not always describe all the relevant symptoms they experience during attacks. One study of patients with probable migraine found more than 90% of patients who did not initially mention light or sound sensitivity acknowledged they preferred a dark and/or quiet room during an attack during follow-up questioning [18]. In addition, patients may present with multiple headache disorders [19,20].

Studies have shown that time constraint is a common concern among health care providers and can lead physicians to ask fewer questions about symptoms related to the presenting complaint [21]. Time constraint may be a particular issue for migraine patients, as studies suggest providers using only closed-ended questions often underestimate migraine severity and impact on patients’ lives [22]. A potential solution to this problem may be to ask the patient to make a separate office visit to address their headache complaint.

### A stepwise approach to the diagnosis of migraine

**Figure 2.**
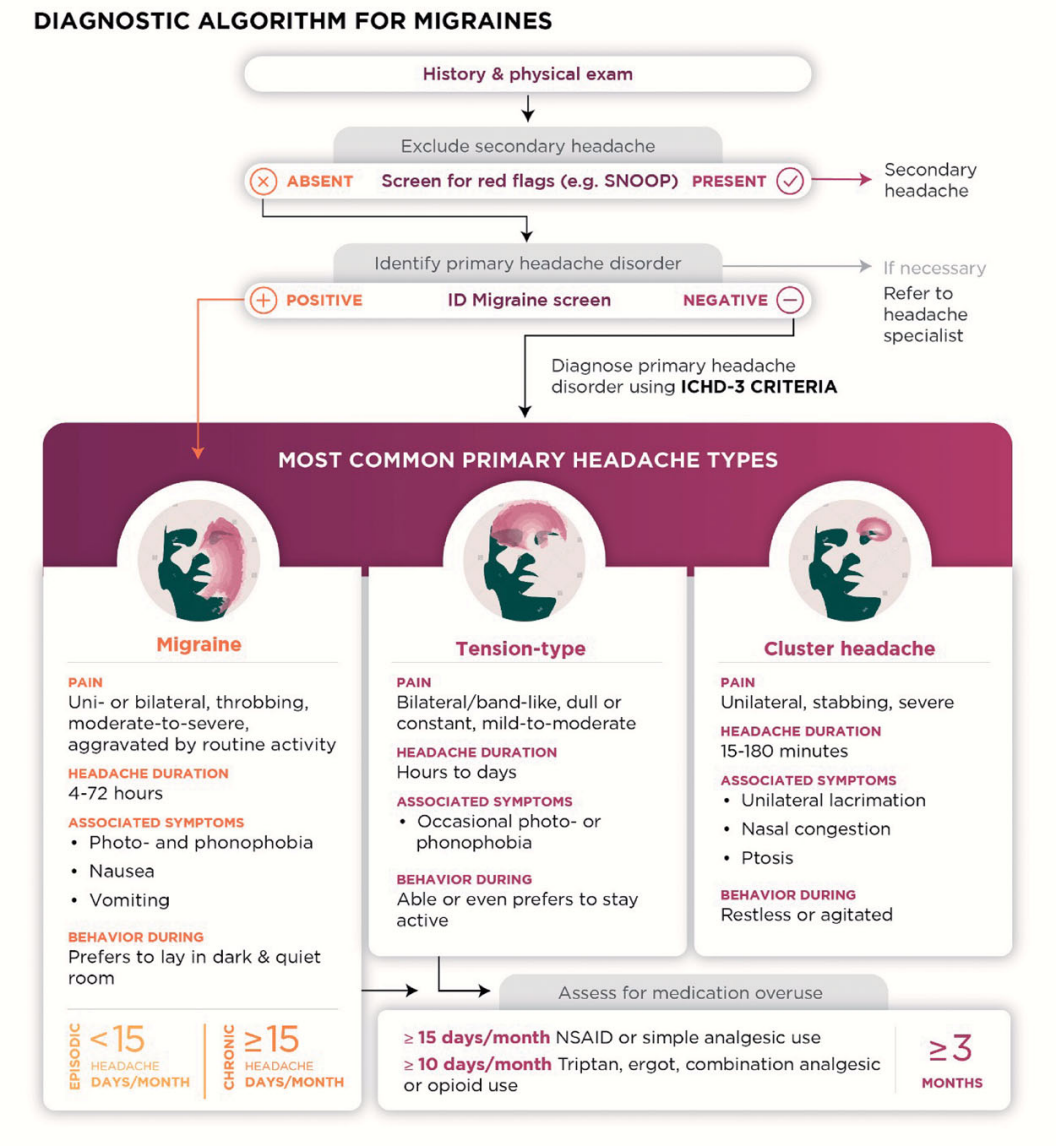
A flow chart for diagnosing migraine in primary care. A headache history and physical exam are the first step in diagnosing migraine, followed by screening for red flags of secondary headache. Likely headache types in primary care can be differentiated by asking about headache duration, features, and frequency. Key headache features for distinguishing migraine, tension-type, and cluster headache are summarised.

In Figure 2, we propose a stepwise approach for diagnosing migraine based on clinical experience and existing literature. To begin, a headache history is the most important tool in diagnosing primary headache disorders, including migraine, while neuroimaging is only required if history or physical examination are suggestive of secondary headache [28]. For patients with multiple types of headaches, a separate history should be taken for each, focussing on the most severe headaches first.

Next, secondary headaches should be ruled out. The updated SNOOP mnemonic (Table 1) can be a useful screening tool for identifying patients with red flags for serious secondary headache disorders [29]. Patients with suspected secondary headache require a diagnostic workup that depends on the red flag identified and the suspected underlying disease, as summarised in Table 1 and reviewed elsewhere [30]. While important to consider, life-threatening secondary headaches–including meningitis, giant cell arteritis, subarachnoid haemorrhage, and malignancy–are very rare among patients presenting with headache in primary care [28].

**Table 1.** The SNOOP4 mnemonic for identifying red flags for secondary headache adapted from [29].

	Sign or symptom	History/Exam features	Associated secondary headache causes*	Potential diagnostic workup*
S	Systemic	History of malignancy, immunosuppression, or HIV Signs of infection (fever, chills, weight loss, etc.)	Infection Malignancy Rheumatic disease Giant cell arteritis	Neuroimaging Lumbar puncture
N	Neurologic	Abnormal neurologic examination Change in behaviour or personality	Malignancy Infection Inflammatory disorder	
O	Onset, sudden	Headache that reaches peak intensity in <1 minute (thunderclap)	Subarachnoid haemorrhage Reversible cerebral vasoconstriction syndromes Stroke	Head CT Lumbar puncture (if CT negative)
O	Older age at onset	New onset headache after age 50	Malignancy Infection Giant cell arteritis	MRI
P	Pattern Change	Change in headache pattern or characteristics progressive headache (loss of headache-free periods)	Malignancy Inflammatory or vascular disorder	
P	Precipitated by Valsalva manoeuvre	Headache precipitated by Valsalva manoeuvre, sneezing, coughing or exercise	Chiari malformation type 1 Posterior fossa lesions Malignancy Arachnoid cysts Subdural haematoma Intracranial hypertension or hypotension	Neuroimaging
P	Postural	Headache precipitated or aggravated by postural change	Intracranial hypertension Intracranial hypotension	Neuroimaging Lumbar puncture MRI with gadolinium (to rule out dural enhancement with suspected CSF leak)
P	Papilledema	Papilledema, visual obscurations, diplopia, or field defects	Intracranial hypertension Malignancy Inflammatory disorder	Thorough funduscopic exam

*Based on clinical experience and [30].

After checking for indicators of secondary headache, the primary headache disorder can be investigated. Validated screening tools such as ID Migraine that focus on the most predictive symptoms–interference with activities, nausea, and sensitivity to light–can be a quick, practical first step to help providers identify patients with migraine [31]. In patients with headache, presence of two of three ID migraine symptoms had a sensitivity of 81%, and a positive predictive value of 93%, for migraine [31].

However, it is important to take a big picture view of the patient’s migraine attack features, rather than focussing on a narrow checklist of symptoms. Using broad, open-ended questions and focussing on the number of headache days (rather than attacks) can help providers fully understand the frequency of and impairment from migraines [22]. Clinical experience suggests it is sometimes necessary to ask patients to follow up with a dedicated headache appointment to ensure enough time for adequate diagnosis.

The main differential diagnoses for migraine are tension-type headache and cluster headache [28]. Distinguishing features between these headache types include the typical location, quality, severity, and duration of pain, associated symptoms, and typical behaviour during attack, as shown in Figure 2 [32]. Briefly, tension headaches generally have mild-to-moderate, bilateral pain and lack migraine-associated symptoms (e.g. nausea and photophobia). Cluster headaches are associated with severe unilateral pain, ipsilateral autonomic symptoms (rhinorrhea, lacrimation, etc.), and duration <3 h. Medication overuse (≥15 days/month for simple analgesics; ≥10 days/month for triptans, ergots, combination analgesics or opioids) can increase the baseline frequency of any headache disorder and should be assessed once the primary headache is diagnosed.

Providers should consider referral to a headache specialist if the diagnosis cannot be confirmed, particularly if secondary headache is suspected [33]. Following diagnosis, referral should be considered if the patient’s quality of life is impaired despite treatment or the patient does not respond to acute therapies [33].

## A contemporary approach to migraine management

It was long believed that migraine was a vascular disease for several reasons, including: the classic throbbing pain of migraine, as well as studies in the mid-twentieth century showing that cerebral vessels were pain sensitive and that vasodilators caused, and vasoconstrictors relieved, headache [34,35]. The vascular theory may have been further bolstered by the effectiveness of triptans, which possess vasoconstrictive activity, for acute migraine relief, and several anti-hypertensive drugs in migraine prevention. Calcitonin gene-related peptide (CGRP), a neuropeptide produced in peripheral sensory neurons and throughout the central nervous system, also has vasodilatory effects which supported the 1985 proposal that it played an important part in migraine pathogenesis [36].

**Figure 3.**
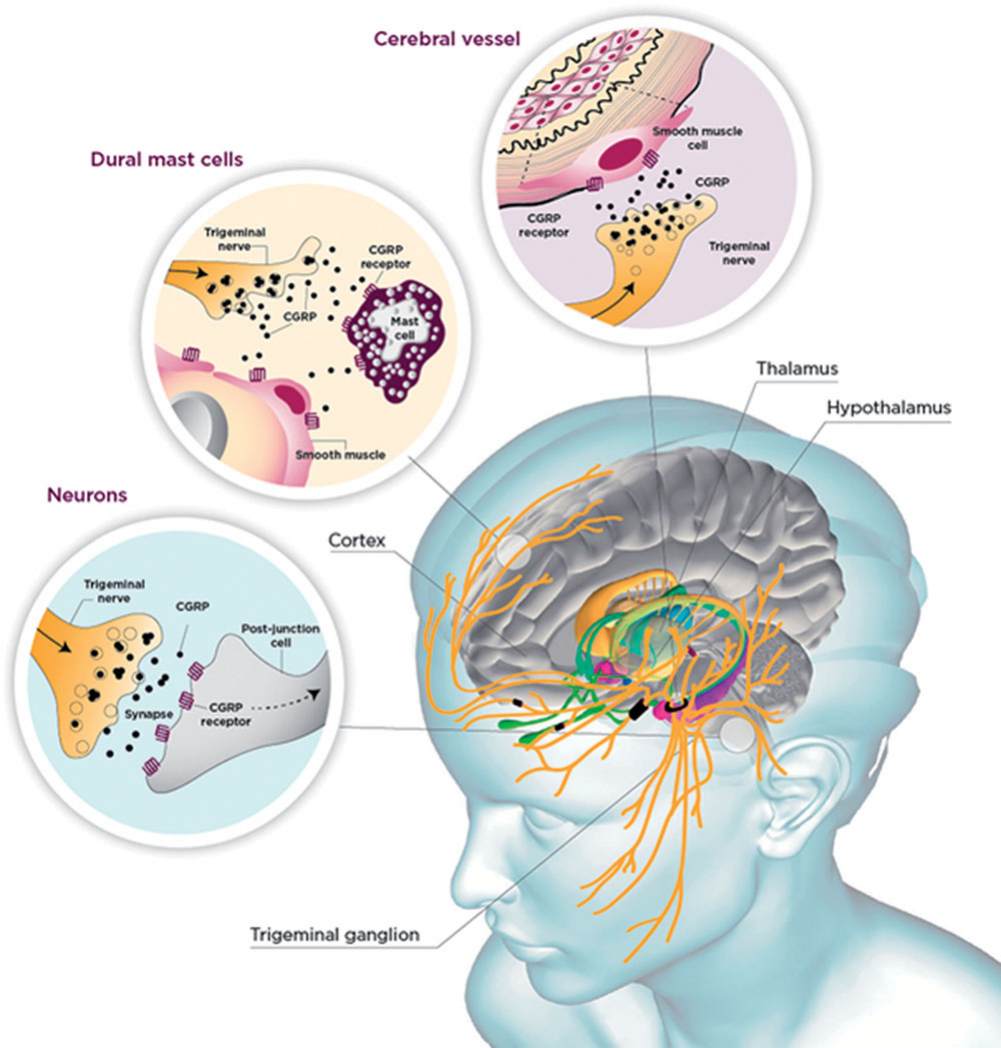
Possible sites of action for CGRP in the pathogenesis of migraine. Neurons in the trigeminal ganglion innervate the face and skull, including the meninges and its vessels. Transmission from the trigeminal ganglion activates second-order neurons in the brain stem, and, in turn, third-order trigeminovascular neurons in the thalamus, which relay nociceptive signals to the cortex resulting in perception of migraine pain [37]. CGRP is released from C fibres from the trigeminal nerve. CGRP receptors are expressed in the smooth muscle of dural blood vessels, by neurons and glia in the trigeminal ganglion, and by some mast cells. Binding of CGRP to its receptor causes activation of trigeminal neurons in the dura and brainstem, vasodilation of dural blood vessels, and release of peptides and cytokines from dural mast cells, which are thought to be part of the cascade of events that occurs with migraine.

CGRP levels were found to be elevated in patient blood, saliva, and cerebrospinal fluid samples during migraine attacks [36]. Follow-up studies showed that intravenous infusion of CGRP triggers migraine-like headaches preferentially in patients with migraine compared to healthy controls, supporting the notion that CGRP may play an important role in migraine pathogenesis [36]. Although the precise function of CGRP during migraine is unknown, CGRP and its receptor are expressed at sites throughout the CNS, as shown in Figure 3, some of which have been linked to migraine symptoms, including pain processing, nausea, photophobia, and phonophobia [36]. Further evidence for the pivotal role of CGRP in migraine comes from clinical trial data showing that multiple pharmacotherapeutics that block the CGRP pathway can effectively manage migraine [36].

The clinical scenarios, advanced neuroimaging data, and experimental neurophysiological findings show that imbalance in inhibitory/excitatory cortical circuits allowing demodulation of subcortical areas is responsible for activation of trigeminovascular system. Hence, demonstrating that activation of trigeminovascular system is not exclusive cause but among main causes of migraine attack [38]. Hemiplegic migraine is a rare subtype of migraine with aura and genetically heterogenous condition. Mutations in the CACNA1A, ATP1A2, and SCN1A genes have been reported to cause these disorders [39]. Many researchers have also indicated the possibility of involvement of PRRT2 gene in migraine pathophysiology. However, further evidence and genetic analyses are required [39–41].

### Strategies to improve patient outcomes

**Figure 4.**
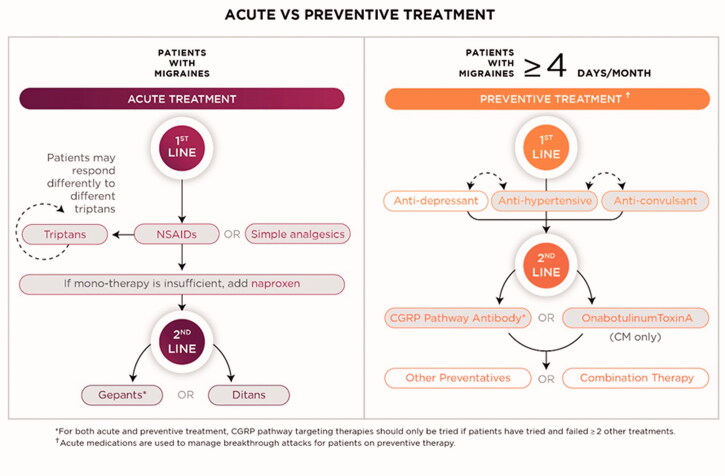
Suggested workflow for migraine management. Patients without contraindications should be offered acute therapy for migraine, starting with NSAIDs (for those with mild-to-moderate symptoms) and triptans. Those who do not respond after appropriate trial periods should be offered another therapy. When migraine interferes with a patient’s quality of life despite acute therapy or patients have more than four migraine days per month, preventive treatment should be offered starting with anti-depressant, anti-hypertensive, or anti-convulsant therapies based on clinical judgement. For both acute and preventive treatment, CGRP pathway targeting therapies should be tried if two or more treatments have failed or are not tolerated. Shaded boxes indicate drug classes with level A evidence.

Effective treatment approaches should consider various factors including patient preferences and comorbidities and should engage patients in setting management goals. The overarching goal of migraine therapy is to improve the patient’s ability to function. Identifying an optimal therapy to reach this goal can be an iterative process for many patients, so it is crucial for providers to help patients set realistic expectations for what successful treatment will look like (i.e. attacks are unlikely to completely cease) and the timeline it may take to reach that end [42]. A thorough understanding of, and accounting for, patient preferences can help providers ensure adherence and treatment continuation [42]. Here, as outlined in Figure 4, we provide a stepwise approach to selecting an appropriate treatment based on the latest American Headache Society (AHS) guidance and our clinical experience.

#### Acute therapy

For acute treatment, the aims include rapid and consistent freedom from pain and other symptoms, return to normal function, minimal need for repeat dosing, and minimal adverse events [42]. According to the AHS guidance and as shown in Figure 4, all patients with migraine should be offered acute therapy; an overview of evidence-based acute treatment options is provided in Table 2. Over-the-counter or prescription NSAIDs and other non-opioid analgesics should be considered first for patients with mild-to-moderate migraine, followed by oral triptans and ergotamines for those who do not get adequate relief, or who have more severe attacks [42]. Of note, while NSAIDs can be appropriate therapy, many individuals will already have tried over-the-counter NSAIDS prior to consulting a provider about headaches. However, prescription NSAIDS may offer an advantage to over-the-couter NSAIDS particularly those that have different formulations (eg. oral powders, intranasal and intramuscular). Triptan non-responders can be switched to a different oral triptan, as this may provide relief to some patients [37]. Although there is evidence that opioids, in particular butorphanol, can be effective for migraine relief, their use is not recommended except in rare instances due to the risk of dependence [43].

**Table 2.** Overview of acute therapeutics for migraine.

Drug	Select examples^a^	Target/ mechanism	Clinical notes^b^
NSAIDs	Aspirin, diclofenac, ibuprofen, naproxen	Cyclooxygenase inhibitor	Caution should be exercised in patients with history of peptic ulcer disease, poorly controlled hypertension, or coronary artery disease
Triptans	Almotriptan, eletriptan, frovatriptan, naratriptan, rizatriptan, sumatriptan, zolmitriptan	Serotonin (5-HT) 1B/D^c^ receptor agonist	Use non-oral routes (nasal spray, subcutaneous) when possible for patients with nausea and/or vomiting Caution should be exercised in patients with poorly controlled hypertension Avoid in patients with hemiplegic migraine, migraine with brainstem aura (“Basilar migraine”) and in patients with history of coronary artery disease/cerebrovascular accident Avoid use of different triptans within 24-hour period
Ergots	DHE	Activates multiple serotonin, noradrenergic, and dopaminergic receptors [32]	Caution should be exercised in patients with poorly controlled hypertension Avoid in patients with hemiplegic migraine, migraine with brainstem aura (“basilar migraine”) and in patients with history of coronary artery disease/cerebrovascular accident Avoid within 24 hours after triptan use
Ditans	Lasmiditan	Serotonin (5-HT) 1F receptor agonist	No contraindication in those with coronary artery disease, cerebrovascular accident, or vascular disease Can cause dizziness; avoid driving for 8 h after dose Schedule V controlled substance (abuse potential)
Gepants	Rimegepant, ubrogepant	CGRP receptor antagonist	No contraindication in those with coronary artery disease, cerebrovascular accident, or vascular disease Avoid with strong (medium for some) CYP3A4 inducers or inhibitors; dose limitations with moderate inhibitors Can be taken on the same day with triptans or ergots

^a^Based on evidence of efficacy (established and probably) [42,43] except for ditans and gepants which are based on current FDA approval.

^b^Information in this column is based on the clinical experience of the authors.

^c^Some triptans also have affinity for the Serotonin (5-HT) 1 F receptor.

AHS currently recommends that patients who have found at least two triptans ineffective or have contraindication to or inability to tolerate triptans should be offered gepants or ditans [42]. The gepants are small molecules that block the CGRP receptor. In clinical trials as acute treatment, two gepants–ubrogepant and rimegepant–were shown to provide more benefit than placebo for freedom from headache pain and other associated symptoms including nausea, photophobia, and phonophobia 2 h after treatment [44,45]. Both gepants had a low incidence of adverse events in clinical trials [44,45].

Ditans are selective serotonin 5-HT_1F_ receptor agonists. In clinical trials as acute treatment, lasmiditan provided more benefit than placebo for freedom from headache pain and other associated symptoms including nausea, photophobia, and phonophobia 2 h after treatment [32]. Gepants and ditans are not thought to cause vasoconstriction and are not contraindicated in patients with cardiovascular disease or cardiovascular risk factors [42]. Acute medication overuse has not been observed with regular use of gepants or ditans. [32].

#### Preventive therapy

For preventive treatment, the aims include reducing the frequency, severity, duration and disability of attacks, reducing reliance on and improving responsiveness to acute treatments, and improving health-related quality of life [42]. Although it can be of high value for patients, preventive treatment is underutilised. In the AMPP study, while almost 40% of individuals with migraine were potential candidates for preventive therapy, only 12% of respondents were currently using a preventive [3]. Prevention is an important strategy if acute treatments are overused–as medication overuse is considered an important contributor to increasing headache frequency and migraine progression [46]–contraindicated, or if patients experience adverse events. Preventive medication should be considered when migraine interferes with a patient’s quality of life despite acute therapy [13], and should be offered to individuals with four or more headache days per month [42].

The order of preference for preventive options is dependent on clinical judgement of patient needs and preferences, evidence of efficacy, tolerability, comorbid and coexisting conditions, and the presence or possibility of pregnancy [42]. An overview of preventive options is provided in Table 3. As shown in Figure 4, first-line, evidence-based preventive therapies include: anti-epileptic drugs (topiramate) and beta-blockers (propranolol, metoprolol, timolol), which have established efficacy, and antidepressants (amitriptyline, venlafaxine) and additional beta-blockers (atenolol, nadolol), which are probably effective [42]. Additional antidepressants (such as nortriptyline and duloxetine) are commonly used for migraine prevention but do not meet the AHS evidence level for established efficacy or probably effective [47]. Although many patients benefit from these therapies, studies have shown that patient adherence to existing oral preventives is low, often because of suboptimal efficacy and poor tolerability [48].

**Table 3.** Overview of preventive therapeutics for migraine.

Drug	Select examples^a^	Administration	Target/ Mechanism	Clinical notes^b^
Anti-hypertensive agents	Atenolol, metoprolol, nadolol, propranolol, timolol,	1–3 times daily, oral	Beta adrenergic receptor antagonist (unknown)	Caution in patients with pre-existing hypotension, poorly controlled depression, asthma/COPD, or diabetes May take 6–8 weeks to notice clinical improvement. Broad therapeutic dosing range, so titrate dose as tolerated
Anti-depressants	Amitriptyline, venlafaxine	Daily, oral	Multiple including: serotonin transporter, norepinephrine transporter, serotonin 5-HT receptors (unknown)	Weight gain, constipation and drowsiness are common May take 6–8 weeks to notice clinical improvement. Broad therapeutic dosing range, so titrate dose as tolerated
Anti-convulsant	Topiramate, valproate sodium	Daily to twice daily, oral	Unknown, possibly related to GABA concentration or GABA receptor activity	Weight gain (sodium valproate) or weight loss (topiramate), drowsiness, and dizziness are common Increased forgetfulness in some patients Avoid in patients who are or may become pregnant May take 6–8 weeks to notice clinical improvement. Broad therapeutic dosing range, so titrate dose as tolerated
OnabotulinumtoxinA		Quarterly, intramuscular	Acetylcholine release inhibitor	Approved for use in CM only Caution in patients with compromised respiratory function, pre-existing neuromuscular disorders Neck pain, headache, worsening migraine, or muscular weakness may occur
CGRP pathway targeting monoclonal antibodies	Eptinezumab	Quarterly, intravenous	Antibody against CGRP	Recommended for patients who have inadequate response to or do not tolerate at least two other preventive agents Hypersensitivity, injection site reactions, new-onset or worsening hypertension, and/or constipation may occur
	Erenumab	Monthly, subcutaneous	Antibody against the CGRP receptor	
	Fremanezumab	Monthly or quarterly, subcutaneous	Antibody against CGRP	
	Galcanezumab	Monthly, subcutaneous	Antibody against CGRP	
Gepants	Rimegepant Atogepant	Every other day, oral for rimegepant and daily for atogepant	CGRP receptor antagonist	No contraindication in those with coronary artery disease, cerebrovascular accident, or vascular disease Avoid with strong (medium for some) CYP3A4 inducers or inhibitors; dose limitations with moderate inhibitors Can be taken on the same day with triptans or ergots

^a^Based on evidence of efficacy (established and probably) [42,43] except for CGRP pathway antibodies and gepants which are based on current FDA approval.

^b^Information in this column is based on the clinical experience of the authors and prescribing information.

OnabotulinumtoxinA has established efficacy for the prevention of CM, but not EM or chronic tension-type headache [42]. In two clinical trials, treatment with onabotulinumtoxinA was found to reduce total headache days in patients with CM with or without acute medication overuse [46]. The mechanism by which onabotulinumtoxinA provides benefit for migraine treatment is thought to involve peripheral inhibition of the release of inflammatory neurotransmitters/peptides [32].

The current AHS guidelines recommend that patients be offered CGRP pathway targeting monoclonal antibodies if they have inadequate response or inability to tolerate ≥2 other preventive treatments (or for CM: ≥2 injections of onabotulinumtoxinA) [42]. Additionally, patients offered CGRP pathway targeting monoclonal antibody therapy should have 4–7 headache days per month with at least moderate disability or ≥8 headache days per month with any disability level [42]. To date, four monoclonal antibodies targeting CGRP or its receptor have shown effectiveness in clinical trials for migraine prevention and are generally well tolerated [49]. Erenumab, fremanezumab, galcanezumab, and eptinezumab showed higher proportions of patients with ≥50% reduction in monthly migraine days relative to placebo for individuals with either EM or CM [37]. Erenumab, fremanezumab, and galcanezumab have shown benefits for patients who had inadequate improvement with other preventive medications [49]. In a five-year, open-label follow-up study of erenumab in EM patients, no new safety signals were observed [50], although reports of constipation with serious complications as well as development and worsening of hypertension for patients taking erenumab were reported through post-marketing surveillance [51].

Recent phase 2/3 clinical trials showed that two oral gepants, rimegepant and atogepant, reduced monthly migraine days compared to placebo when taken as preventive therapy [52,53]. Rimegepant and atogepant were recently approved by the Food and Drug Administration (FDA) for migraine prevention in the United States. Rimegepant has an every other day dosing regimen and is the first medication approved for use as both an acute and preventive treatment for migraine[54]. I Atogepant has a daily dosing regimen and was approved for preventive use only. 

One major differentiating factor between the migraine preventives is the route of administration and dosing schedule (Table 3). OnabotulinumtoxinA is administered intramuscularly once per quarter by a heath care provider. Among the CGRP pathway targeting monoclonal antibodies, eptinezumab is a quarterly, intravenous administration by a heath care provider, while the remaining therapies are taken monthly (fremanezumab can be administered monthly or quarterly) and can be self-administered. Established oral preventives are administered daily or multiple times per day. The less frequent dosing required for injectable options may be helpful for patients who have struggled with medication adherence. However, some patients may find injectable options unappealing compared to pills. Overall, potential advantages of injectable options compared to the oral preventives include low side effect profiles, minimal drug-drug interactions, and no need for gradual dose escalation or titration [42].

With all treatments, it is important to evaluate the efficacy and tolerability of the medication, and consider changes in dose, adding therapeutics, or changing treatments, if appropriate. AHS guidelines suggest efficacy trial periods of at least 8 weeks at the target dose for oral preventives, 3–6 months for monoclonal antibodies, and 6 months for onabotulinumtoxinA, while acute medications should be tried for at least two attacks [42]. Notably, patients may value treatment outcomes differently; for example, increasing functional ability and quality of life may mean more to patients than the 50% reduction in monthly headache days that is often measured in clinical trials [42].

#### Non-Pharmacological approach

The non-pharmacological management of migraine includes lifestyle modification and identifying the triggers and avoiding or managing them to prevent or delay progression of migraine [4,55]. Physical activity and sports have a protective effect in patients with migraine [6,7]. Currently, there are no of evidence-based dietary recommendations available for patients with migraine, however unhealthy food habits are always a risk factor [7,55]. The chronification of migraine and the number of migraine attacks can be prevented by improving sleep quality or treating sleep disorder along with migraine [7]. Direct link between decrease in physical activity and worsening sleep was observed [7]. In uncommon conditions, such as hemiplegic migraine avoiding triggers like emotional and physical stress, viral infection and head trauma are part of managing the condition [39].

## Concluding remarks and future directions

Migraine is a widespread disease that causes impairment and disability for tens of millions of Americans. Despite its prevalence, migraine is underdiagnosed and undertreated. Optimising expeditious diagnosis and appropriate treatment can lessen patient suffering and may minimise disease progression for some individuals.

Internists and other primary care providers are key to achieving timely diagnosis and effective treatment for many migraine patients, the majority of whom are seen in primary care settings. Providers can improve their recognition of migraine by understanding the variability of migraine-associated symptoms, utilising diagnostic screening tools, having a comprehensive view of migraine diagnostic criteria, and taking a thorough headache history. Once patients are diagnosed with migraine, shared decision making can be an important approach to effective migraine management. This can include initiating abortive treatments for acute attacks, educating patients about the risks of overusing acute therapies, early initiation of preventive treatment when appropriate, and incorporating patient preferences and co-existing conditions into migraine care strategies.

As our understanding of migraine pathophysiology expands and new therapies arrive in the clinic, the optimal treatment approach will continue to evolve. It is crucial to adopt a rational approach to the diagnosis and management of migraine to reduce the burden of this common and debilitating disease, and to improve the lives of patients and their families.

## Data Availability

Data sharing is not applicable to this article as no new data were created or analysed in this study.

## Abbreviations

5-HT: 5-hydroxytryptamine
AHS: American Headache Society
AMPP: American Migraine Prevalence and Prevention
CGRP: calcitonin gene related peptide
CM: chronic migraine
CNS: central nervous system
COPD: chronic obstructive pulmonary disease
CSF: cerebrospinal fluid
CT: computed tomography
CYP3A4: cytochrome P450 3A4
EM: episodic migraine
HIV: human immunodeficiency virus
ICD-9: International Classification of Diseases, Ninth Revision
ICHD-3: International Classification of Headache Disorders, third edition
MRI: magnetic resonance imaging
NSAID: non-steroidal anti-inflammatory drug

## References

[1] Stovner LJ, Nichols E, Steiner TJ, et al. Global, regional, and national burden of migraine and tension-type headache, 1990–2016: a systematic analysis for the global burden of disease study 2016. Lancet Neurol. 2018;17(11):954–976. 30353868 10.1016/S1474-4422(18)30322-3 PMC6191530

[2] Steiner TJ, Stovner LJ, Vos T, et al. Migraine is first cause of disability in under 50s: will health politicians now take notice? J Headache Pain. 2018;19(1):17. 29468450 10.1186/s10194-018-0846-2 PMC5821623

[3] Lipton RB, Bigal ME, Diamond M, et al. Migraine prevalence, disease burden, and the need for preventive therapy. Neurology. 2007;68(5):343–349. 17261680 10.1212/01.wnl.0000252808.97649.21

[4] Marmura MJ. Triggers, protectors, and predictors in episodic migraine. Curr Pain Headache Rep. 2018;22(12):81. 30291562 10.1007/s11916-018-0734-0

[5] Andress-Rothrock D, King W, Rothrock J. An analysis of migraine triggers in a clinic-based population. Headache. 2010;50(8):1366–1370. 21044280 10.1111/j.1526-4610.2010.01753.x

[6] Pilati L, Battaglia G, Di Stefano V, et al. Migraine and sport in a physically active population of students: results of a cross-sectional study. Headache. 2020;60(10):2330–2339. 33159460 10.1111/head.14015

[7] Di Stefano V, Ornello R, Gagliardo A, et al. Social distancing in chronic migraine during the COVID-19 outbreak: results from a multicenter observational study. Nutrients. 2021;13(4):1361. 33921674 10.3390/nu13041361 PMC8074143

[8] Finley CR, Chan DS, Garrison S, et al. What are the most common conditions in primary care? Systematic review. Can Fam Physician. 2018;64(11):832–840. 30429181 PMC6234945

[9] Peabody MR, O'Neill TR, Stelter KL, et al. Frequency and criticality of diagnoses in family medicine practices: from the national ambulatory medical care survey (NAMCS). J Am Board Fam Med. 2018;31(1):126–138. 29330247 10.3122/jabfm.2018.01.170209

[10] Headache classification committee of the international headache society (IHS) the international classification of headache disorders. Cephalalgia. 2018;38(1):1–211. 10.1177/0333102417738202 29368949

[11] Tepper SJ, Dahlof CG, Dowson A, et al. Prevalence and diagnosis of migraine in patients consulting their physician with a complaint of headache: data from the landmark study. Headache. 2004;44(9):856–864. 15447694 10.1111/j.1526-4610.2004.04167.x

[12] Martin VT TR, Al-Shaikh E, Martin AT, Levin LS, editors. Comorbid medical disorders and migraine: does gender make a difference? International headache congress. Boston (MA): Cephalalgia; 2013.

[13] Becker WJ, Findlay T, Moga C, et al. Guideline for primary care management of headache in adults. Can Fam Physician. 2015;61(8):670–679. 26273080 PMC4541429

[14] Dodick DW, Loder EW, Manack Adams A, et al. Assessing barriers to chronic migraine consultation, diagnosis, and treatment: Results from the chronic migraine epidemiology and outcomes (CaMEO) study. Headache. 2016;56(5):821–834. 27143127 10.1111/head.12774 PMC5084794

[15] Lipton RB, Serrano D, Holland S, et al. Barriers to the diagnosis and treatment of migraine: effects of sex, income, and headache features. Headache. 2013;53(1):81–92. 23078241 10.1111/j.1526-4610.2012.02265.x

[16] Viana M, Sances G, Ghiotto N, et al. Variability of the characteristics of a migraine attack within patients. Cephalalgia. 2016;36(9):825–830. 26498348 10.1177/0333102415613612

[17] Vollesen AL, Benemei S, Cortese F, et al. Migraine and cluster headache – the common link. J Headache Pain. 2018;19(1):89. 30242519 10.1186/s10194-018-0909-4 PMC6755613

[18] Evans RW, Seifert T, Kailasam J, et al. The use of questions to determine the presence of photophobia and phonophobia during migraine. Headache. 2008;48(3):395–397. 17868350 10.1111/j.1526-4610.2007.00920.x

[19] Yoon MS, Katsarava Z, Obermann M, et al. Prevalence of primary headaches in Germany: results of the German Headache Consortium study. J Headache Pain. 2012;13(3):215–223. 22395638 10.1007/s10194-012-0425-x PMC3311829

[20] Stang PE, Von Korff M. The diagnosis of headache in primary care: factors in the agreement of clinical and standardized diagnoses. Headache. 1994;34(3):138–142. 8200786 10.1111/j.1526-4610.1994.hed3403138.x

[21] Tsiga E, Panagopoulou E, Sevdalis N, et al. The influence of time pressure on adherence to guidelines in primary care: an experimental study. BMJ Open. 2013;3(4):e002700. 10.1136/bmjopen-2013-002700 PMC3641486 23585394

[22] Lipton RB, Hahn SR, Cady RK, et al. In-office discussions of migraine: results from the American Migraine Communication study. J Gen Intern Med. 2008;23(8):1145–1151. 18459012 10.1007/s11606-008-0591-3 PMC2517978

[23] Evans RW. The clinical features of migraine with and without aura. Practical Neurology. 2014;14:26–32.

[24] Lai TH, Fuh JL, Wang SJ. Cranial autonomic symptoms in migraine: characteristics and comparison with cluster headache. J Neurol Neurosurg Psychiatry. 2009;80(10):1116–1119. 18931007 10.1136/jnnp.2008.157743

[25] Kelman L. Migraine pain location: a tertiary care study of 1283 migraineurs. Headache. 2005;45(8):1038–1047. 16109118 10.1111/j.1526-4610.2005.05185.x

[26] Silberstein SD. Migraine symptoms: results of a survey of self-reported migraineurs. Headache. 1995;35(7):387–396. 7672955 10.1111/j.1526-4610.1995.hed3507387.x

[27] Martins IP, Gouveia RG, Parreira E. Kinesiophobia in migraine. J Pain. 2006;7(6):445–451. 16750801 10.1016/j.jpain.2006.01.449

[28] Steiner TJ, Jensen R, Katsarava Z, et al. Aids to management of headache disorders in primary care (2nd edition): on behalf of the European Headache Federation and Lifting the Burden: the Global Campaign against Headache. J Headache Pain. 2019;20(1):57. 31113373 10.1186/s10194-018-0899-2 PMC6734476

[29] Dodick DW. Pearls: headache. Semin Neurol. 2010;30(1):74–81. 20127586 10.1055/s-0029-1245000

[30] Chiang C-C, VanderPluym J. Diagnosing secondary headaches. Practical Neurology. 2020;20.

[31] Lipton RB, Dodick D, Sadovsky R, et al. A self-administered screener for migraine in primary care: the ID migraine validation study. Neurology. 2003;61(3):375–382. 12913201 10.1212/01.wnl.0000078940.53438.83

[32] Robbins MS. Diagnosis and management of headache: a review. JAMA. 2021;325(18):1874–1885. 33974014 10.1001/jama.2021.1640

[33] Beithon J, Gallenberg M, Johnson K, et al. Diagnosis and Treatment of Headache: Healthcare Guideline Bloomington, MN: Institute for Clinical Systems Improvement; 2013. [11th]. Available from: https://www.icsi.org/wp-content/uploads/2019/01/Headache.pdf

[34] Brennan KC, Charles A. An update on the blood vessel in migraine. Curr Opin Neurol. 2010;23(3):266–274. 20216215 10.1097/WCO.0b013e32833821c1 PMC5500293

[35] Ahn AH. Southern headache society supplement: the neurobiology of throbbing pain in migraine. Headache. 2012;52(Suppl 1):12–14. 22540199 10.1111/j.1526-4610.2012.02136.x PMC3349965

[36] Edvinsson L, Haanes KA, Warfvinge K, et al. CGRP as the target of new migraine therapies – successful translation from bench to clinic. Nat Rev Neurol. 2018;14(6):338–350. 29691490 10.1038/s41582-018-0003-1

[37] Ashina M. Migraine. N Engl J Med. 2020;383(19):1866–1876. 33211930 10.1056/NEJMra1915327

[38] Barbanti P, Brighina F, Egeo G, et al. Migraine as a cortical brain disorder. Headache. 2020;60(9):2103–2114. 32851650 10.1111/head.13935

[39] Di Stefano V, Rispoli MG, Pellegrino N, et al. Diagnostic and therapeutic aspects of hemiplegic migraine. J Neurol Neurosurg Psychiatry. 2020;91(7):764–771. 32430436 10.1136/jnnp-2020-322850 PMC7361005

[40] Sheerin UM, Stamelou M, Charlesworth G, et al. Migraine with aura as the predominant phenotype in a family with a PRRT2 mutation. J Neurol. 2013;260(2):656–660. 23180180 10.1007/s00415-012-6747-4 PMC4193291

[41] de Boer I, van den Maagdenberg A, Terwindt GM. Advance in genetics of migraine. Curr Opin Neurol. 2019;32(3):413–421. 30883436 10.1097/WCO.0000000000000687 PMC6522206

[42] Ailani J, Burch RC, Robbins MS. The American Headache Society Consensus statement: update on integrating new migraine treatments into clinical practice. Headache. 2021;61(7):1021–1039. 34160823 10.1111/head.14153

[43] Marmura MJ, Silberstein SD, Schwedt TJ. The acute treatment of migraine in adults: the American Headache Society Evidence assessment of migraine pharmacotherapies. Headache. 2015;55(1):3–20. 25600718 10.1111/head.12499

[44] Croop R, Goadsby PJ, Stock DA, et al. Efficacy, safety, and tolerability of rimegepant orally disintegrating tablet for the acute treatment of migraine: a randomised, phase 3, double-blind, placebo-controlled trial. Lancet. 2019;394(10200):737–745. 31311674 10.1016/S0140-6736(19)31606-X

[45] Lipton RB, Dodick DW, Ailani J, et al. Effect of ubrogepant vs placebo on pain and the most bothersome associated symptom in the acute treatment of migraine: the ACHIEVE II randomized clinical trial. JAMA. 2019;322(19):1887–1898. 31742631 10.1001/jama.2019.16711 PMC6865323

[46] May A, Schulte LH. Chronic migraine: risk factors, mechanisms and treatment. Nat Rev Neurol. 2016;12(8):455–464. 27389092 10.1038/nrneurol.2016.93

[47] Burch R. Antidepressants for preventive treatment of migraine. Curr Treat Options Neurol. 2019;21(4):18. 30895388 10.1007/s11940-019-0557-2

[48] Blumenfeld AM, Bloudek LM, Becker WJ, et al. Patterns of use and reasons for discontinuation of prophylactic medications for episodic migraine and chronic migraine: results from the second international burden of migraine study (IBMS-II). Headache. 2013;53(4):644–655. 23458496 10.1111/head.12055

[49] Charles A, Pozo-Rosich P. Targeting calcitonin gene-related peptide: a new era in migraine therapy. Lancet. 2019;394(10210):1765–1774. 31668411 10.1016/S0140-6736(19)32504-8

[50] Ashina M, Goadsby PJ, Reuter U, et al. Long-term efficacy and safety of erenumab in migraine prevention: Results from a 5-year, open-label treatment phase of a randomized clinical trial. Eur J Neurol. 2021;28(5):1716–1725. 33400330 10.1111/ene.14715 PMC8248354

[51] Aimovig (erenumab-aooe) [package insert]. Thousand Oaks, CA: Amgen Inc. 2021.

[52] Goadsby PJ, Dodick DW, Ailani J, et al. Safety, tolerability, and efficacy of orally administered atogepant for the prevention of episodic migraine in adults: a double-blind, randomised phase 2b/3 trial. Lancet Neurol. 2020;19(9):727–737. 32822633 10.1016/S1474-4422(20)30234-9

[53] Croop R, Lipton RB, Kudrow D, et al. Oral rimegepant for preventive treatment of migraine: a phase 2/3, randomised, double-blind, placebo-controlled trial. Lancet. 2021;397(10268):51–60. 33338437 10.1016/S0140-6736(20)32544-7

[54] Nurtect ODT (rimegepant) [package insert]. New Haven (CT): Biohaven Pharmaceuticals Inc.; 2021.

[55] Gazerani P. Migraine and diet. Nutrients. 2020;12(6):1658. 32503158 10.3390/nu12061658 PMC7352457

